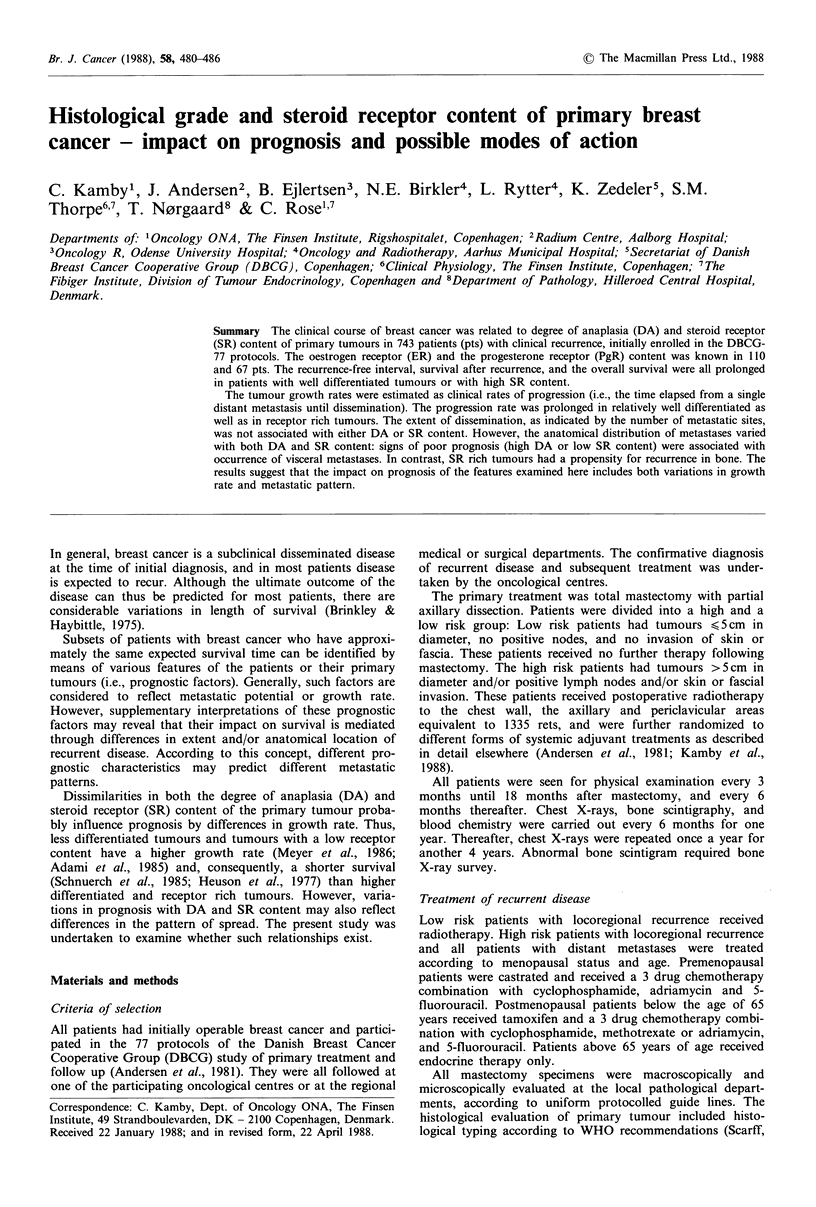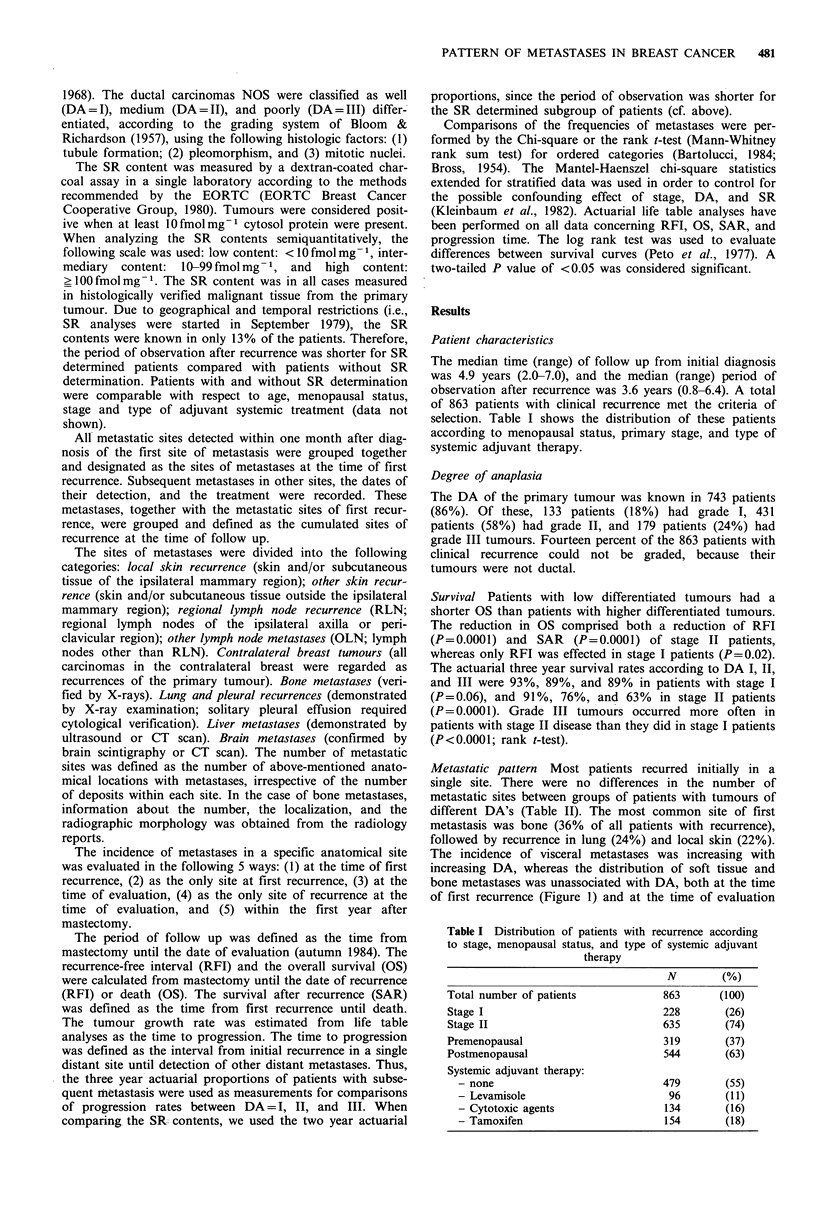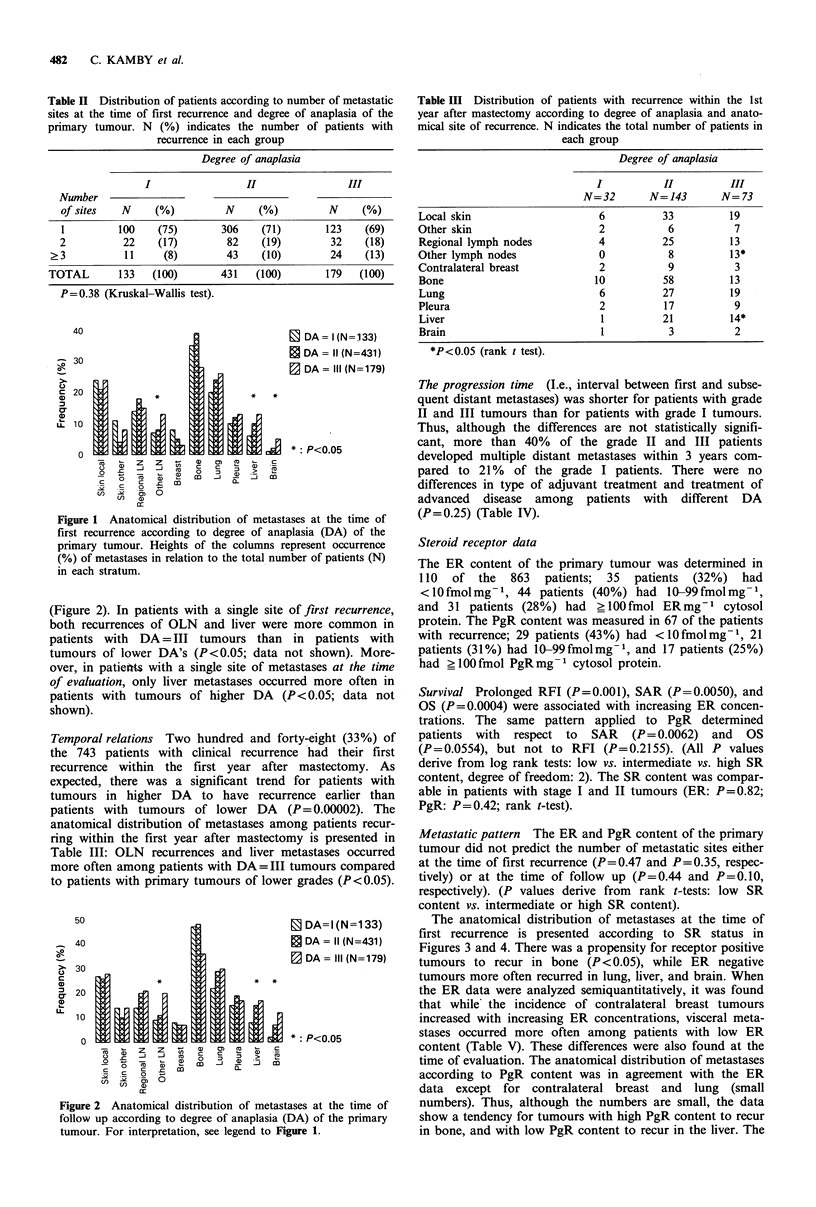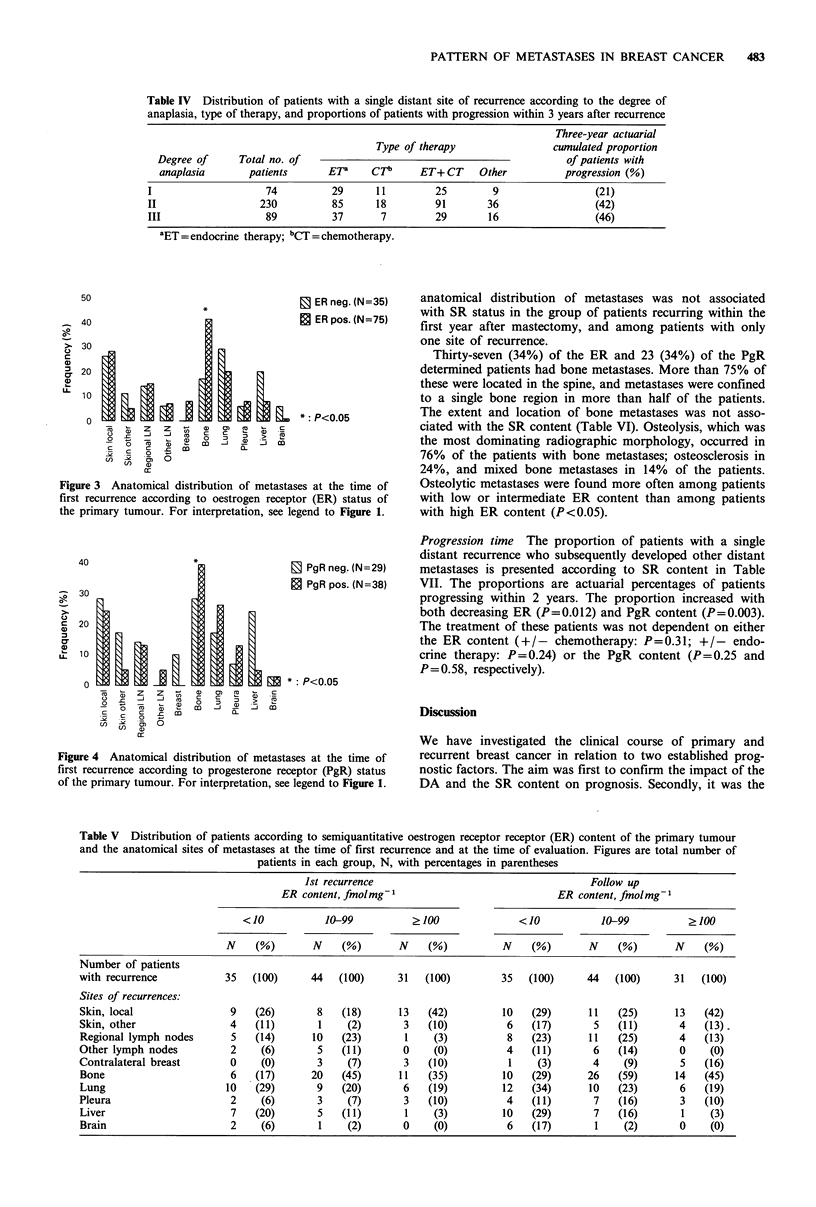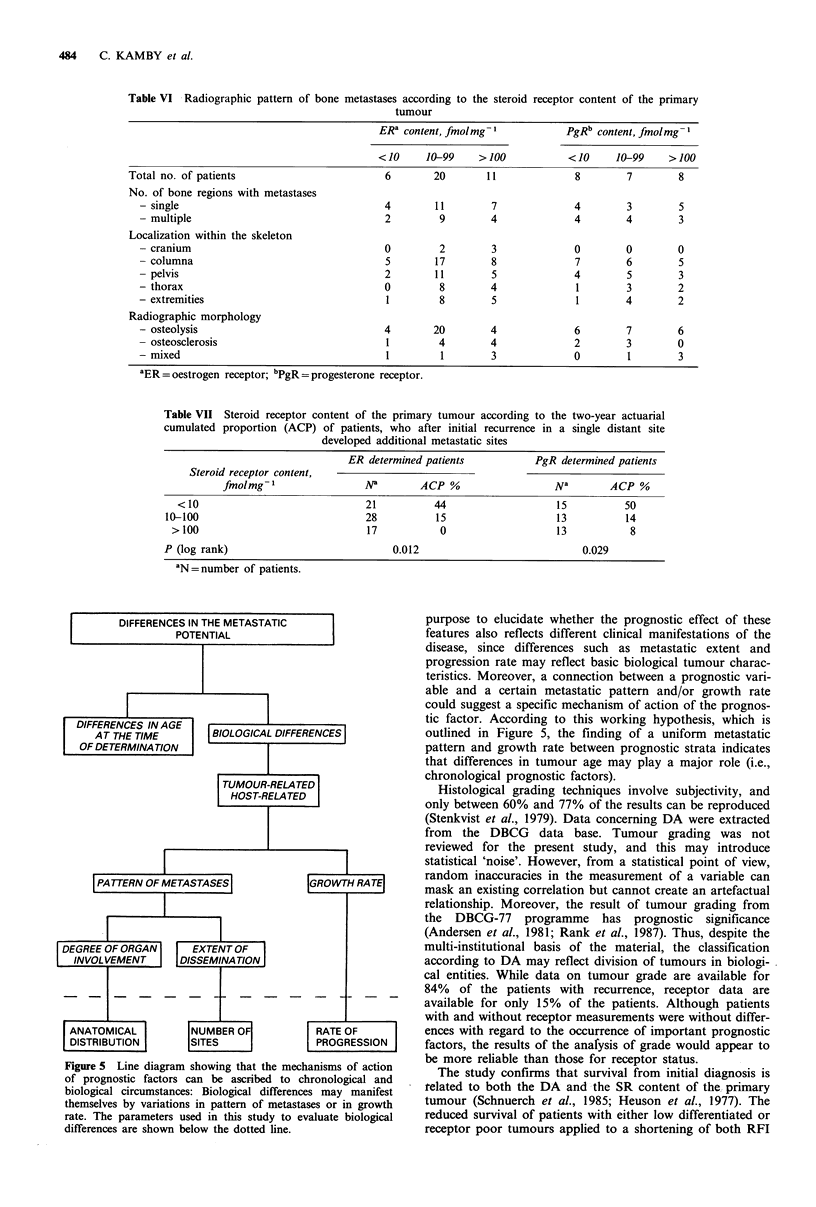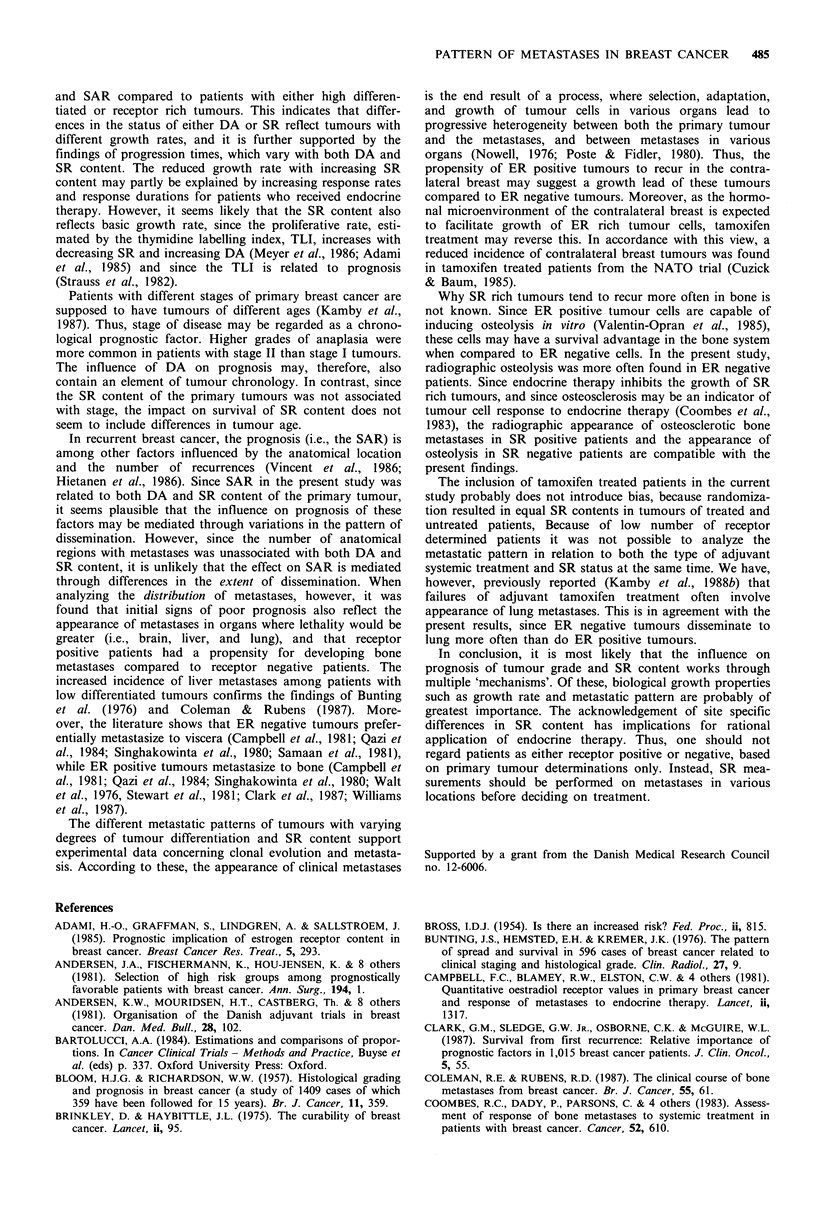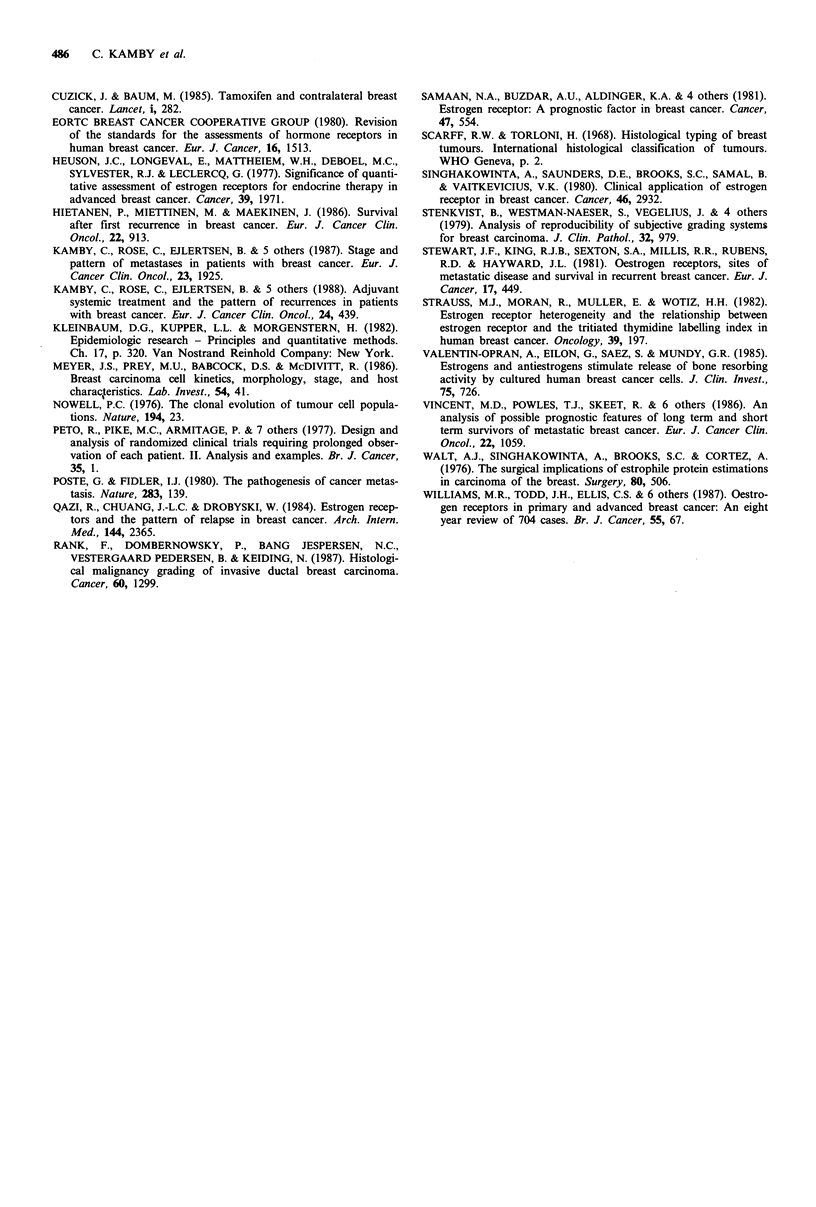# Histological grade and steroid receptor content of primary breast cancer--impact on prognosis and possible modes of action.

**DOI:** 10.1038/bjc.1988.245

**Published:** 1988-10

**Authors:** C. Kamby, J. Andersen, B. Ejlertsen, N. E. Birkler, L. Rytter, K. Zedeler, S. M. Thorpe, T. NÃ¸rgaard, C. Rose

**Affiliations:** Department of Oncology, Finsen Institute, Rigshospitalet, Copenhagen, Denmark.

## Abstract

The clinical course of breast cancer was related to degree of anaplasia (DA) and steroid receptor (SR) content of primary tumours in 743 patients (pts) with clinical recurrence, initially enrolled in the DBCG-77 protocols. The oestrogen receptor (ER) and the progesterone receptor (PgR) content was known in 110 and 67 pts. The recurrence-free interval, survival after recurrence, and the overall survival were all prolonged in patients with well differentiated tumours or with high SR content. The tumour growth rates were estimated as clinical rates of progression (i.e., the time elapsed from a single distant metastasis until dissemination). The progression rate was prolonged in relatively well differentiated as well as in receptor rich tumours. The extent of dissemination, as indicated by the number of metastatic sites, was not associated with either DA or SR content. However, the anatomical distribution of metastases varied with both DA and SR content: signs of poor prognosis (high DA or low SR content) were associated with occurrence of visceral metastases. In contrast, SR rich tumours had a propensity for recurrence in bone. The results suggest that the impact on prognosis of the features examined here includes both variations in growth rate and metastatic pattern.


*Br. J. Cancer* (1988), **58**, 480–486 © The Macmillan Press Ltd., 1988

### Histological grade and steroid receptor content of primary breast cancer – impact on prognosis and possible modes of action

C. Kamby[1], J. Andersen[2], B. Ejlertsen[3], N.E. Birkler[4], L. Rytter[4], K. Zedeler[5], S.M. Thorpe[6,7], T. Nørgaard[8] & C. Rose[1,7]

*Departments of: [1]Oncology ONA, The Finsen Institute, Rigshospitalet, Copenhagen; [2]Radium Centre, Aalborg Hospital; [3]Oncology R, Odense University Hospital; [4]Oncology and Radiotherapy, Aarhus Municipal Hospital; [5]Secretariat of Danish Breast Cancer Cooperative Group (DBCG), Copenhagen; [6]Clinical Physiology, The Finsen Institute, Copenhagen; [7]The Fibiger Institute, Division of Tumour Endocrinology, Copenhagen and [8]Department of Pathology, Hilleroed Central Hospital, Denmark.*

**Summary** The clinical course of breast cancer was related to degree of anaplasia (DA) and steroid receptor (SR) content of primary tumours in 743 patients (pts) with clinical recurrence, initially enrolled in the DBCG-77 protocols. The oestrogen receptor (ER) and the progesterone receptor (PgR) content was known in 110 and 67 pts. The recurrence-free interval, survival after recurrence, and the overall survival were all prolonged in patients with well differentiated tumours or with high SR content.

The tumour growth rates were estimated as clinical rates of progression (i.e., the time elapsed from a single distant metastasis until dissemination). The progression rate was prolonged in relatively well differentiated as well as in receptor rich tumours. The extent of dissemination, as indicated by the number of metastatic sites, was not associated with either DA or SR content. However, the anatomical distribution of metastases varied with both DA and SR content: signs of poor prognosis (high DA or low SR content) were associated with occurrence of visceral metastases. In contrast, SR rich tumours had a propensity for recurrence in bone. The results suggest that the impact on prognosis of the features examined here includes both variations in growth rate and metastatic pattern.

In general, breast cancer is a subclinical disseminated disease at the time of initial diagnosis, and in most patients disease is expected to recur. Although the ultimate outcome of the disease can thus be predicted for most patients, there are considerable variations in length of survival (Brinkley & Haybittle, 1975).

Subsets of patients with breast cancer who have approximately the same expected survival time can be identified by means of various features of the patients or their primary tumours (i.e., prognostic factors). Generally, such factors are considered to reflect metastatic potential or growth rate. However, supplementary interpretations of these prognostic factors may reveal that their impact on survival is mediated through differences in extent and/or anatomical location of recurrent disease. According to this concept, different prognostic characteristics may predict different metastatic patterns.

Dissimilarities in both the degree of anaplasia (DA) and steroid receptor (SR) content of the primary tumour probably influence prognosis by differences in growth rate. Thus, less differentiated tumours and tumours with a low receptor content have a higher growth rate (Meyer *et al.*, 1986; Adami *et al.*, 1985) and, consequently, a shorter survival (Schnuerch *et al.*, 1985; Heuson *et al.*, 1977) than higher differentiated and receptor rich tumours. However, variations in prognosis with DA and SR content may also reflect differences in the pattern of spread. The present study was undertaken to examine whether such relationships exist.

### Materials and methods

### *Criteria of selection*

All patients had initially operable breast cancer and participated in the 77 protocols of the Danish Breast Cancer Cooperative Group (DBCG) study of primary treatment and follow up (Andersen *et al.*, 1981). They were all followed at one of the participating oncological centres or at the regional medical or surgical departments. The confirmative diagnosis of recurrent disease and subsequent treatment was undertaken by the oncological centres.

The primary treatment was total mastectomy with partial axillary dissection. Patients were divided into a high and a low risk group: Low risk patients had tumours $\leqslant 5$ cm in diameter, no positive nodes, and no invasion of skin or fascia. These patients received no further therapy following mastectomy. The high risk patients had tumours $>5$ cm in diameter and/or positive lymph nodes and/or skin or fascial invasion. These patients received postoperative radiotherapy to the chest wall, the axillary and periclavicular areas equivalent to 1335 rets, and were further randomized to different forms of systemic adjuvant treatments as described in detail elsewhere (Andersen *et al.*, 1981; Kamby *et al.*, 1988).

All patients were seen for physical examination every 3 months until 18 months after mastectomy, and every 6 months thereafter. Chest X-rays, bone scintigraphy, and blood chemistry were carried out every 6 months for one year. Thereafter, chest X-rays were repeated once a year for another 4 years. Abnormal bone scintigram required bone X-ray survey.

### *Treatment of recurrent disease*

Low risk patients with locoregional recurrence received radiotherapy. High risk patients with locoregional recurrence and all patients with distant metastases were treated according to menopausal status and age. Premenopausal patients were castrated and received a 3 drug chemotherapy combination with cyclophosphamide, adriamycin and 5-fluorouracil. Postmenopausal patients below the age of 65 years received tamoxifen and a 3 drug chemotherapy combination with cyclophosphamide, methotrexate or adriamycin, and 5-fluorouracil. Patients above 65 years of age received endocrine therapy only.

All mastectomy specimens were macroscopically and microscopically evaluated at the local pathological departments, according to uniform protocolled guide lines. The histological evaluation of primary tumour included histological typing according to WHO recommendations (Scarff,

Correspondence: C. Kamby, Dept. of Oncology ONA, The Finsen Institute, 49 Strandboulevarden, DK – 2100 Copenhagen, Denmark.
Received 22 January 1988; and in revised form, 22 April 1988.

1968). The ductal carcinomas NOS were classified as well (DA = I), medium (DA = II), and poorly (DA = III) differentiated, according to the grading system of Bloom & Richardson (1957), using the following histologic factors: (1) tubule formation; (2) pleomorphism, and (3) mitotic nuclei.

The SR content was measured by a dextran-coated charcoal assay in a single laboratory according to the methods recommended by the EORTC (EORTC Breast Cancer Cooperative Group, 1980). Tumours were considered positive when at least 10 fmol mg$^{-1}$ cytosol protein were present. When analyzing the SR contents semiquantitatively, the following scale was used: low content: < 10 fmol mg$^{-1}$, intermediary content: 10–99 fmol mg$^{-1}$, and high content: ≧ 100 fmol mg$^{-1}$. The SR content was in all cases measured in histologically verified malignant tissue from the primary tumour. Due to geographical and temporal restrictions (i.e., SR analyses were started in September 1979), the SR contents were known in only 13% of the patients. Therefore, the period of observation after recurrence was shorter for SR determined patients compared with patients without SR determination. Patients with and without SR determination were comparable with respect to age, menopausal status, stage and type of adjuvant systemic treatment (data not shown).

All metastatic sites detected within one month after diagnosis of the first site of metastasis were grouped together and designated as the sites of metastases at the time of first recurrence. Subsequent metastases in other sites, the dates of their detection, and the treatment were recorded. These metastases, together with the metastatic sites of first recurrence, were grouped and defined as the cumulated sites of recurrence at the time of follow up.

The sites of metastases were divided into the following categories: *local skin recurrence* (skin and/or subcutaneous tissue of the ipsilateral mammary region); *other skin recurrence* (skin and/or subcutaneous tissue outside the ipsilateral mammary region); *regional lymph node recurrence* (RLN; regional lymph nodes of the ipsilateral axilla or periclavicular region); *other lymph node metastases* (OLN; lymph nodes other than RLN). *Contralateral breast tumours* (all carcinomas in the contralateral breast were regarded as recurrences of the primary tumour). *Bone metastases* (verified by X-rays). *Lung and pleural recurrences* (demonstrated by X-ray examination; solitary pleural effusion required cytological verification). *Liver metastases* (demonstrated by ultrasound or CT scan). *Brain metastases* (confirmed by brain scintigraphy or CT scan). The number of metastatic sites was defined as the number of above-mentioned anatomical locations with metastases, irrespective of the number of deposits within each site. In the case of bone metastases, information about the number, the localization, and the radiographic morphology was obtained from the radiology reports.

The incidence of metastases in a specific anatomical site was evaluated in the following 5 ways: (1) at the time of first recurrence, (2) as the only site at first recurrence, (3) at the time of evaluation, (4) as the only site of recurrence at the time of evaluation, and (5) within the first year after mastectomy.

The period of follow up was defined as the time from mastectomy until the date of evaluation (autumn 1984). The recurrence-free interval (RFI) and the overall survival (OS) were calculated from mastectomy until the date of recurrence (RFI) or death (OS). The survival after recurrence (SAR) was defined as the time from first recurrence until death. The tumour growth rate was estimated from life table analyses as the time to progression. The time to progression was defined as the interval from initial recurrence in a single distant site until detection of other distant metastases. Thus, the three year actuarial proportions of patients with subsequent metastasis were used as measurements for comparisons of progression rates between DA = I, II, and III. When comparing the SR contents, we used the two year actuarial proportions, since the period of observation was shorter for the SR determined subgroup of patients (cf. above).

Comparisons of the frequencies of metastases were performed by the Chi-square or the rank $t$-test (Mann-Whitney rank sum test) for ordered categories (Bartolucci, 1984; Bross, 1954). The Mantel-Haenszel chi-square statistics extended for stratified data was used in order to control for the possible confounding effect of stage, DA, and SR (Kleinbaum *et al.*, 1982). Actuarial life table analyses have been performed on all data concerning RFI, OS, SAR, and progression time. The log rank test was used to evaluate differences between survival curves (Peto *et al.*, 1977). A two-tailed $P$ value of $<0.05$ was considered significant.

### Results

### *Patient characteristics*

The median time (range) of follow up from initial diagnosis was 4.9 years (2.0–7.0), and the median (range) period of observation after recurrence was 3.6 years (0.8–6.4). A total of 863 patients with clinical recurrence met the criteria of selection. Table I shows the distribution of these patients according to menopausal status, primary stage, and type of systemic adjuvant therapy.

### *Degree of anaplasia*

The DA of the primary tumour was known in 743 patients (86%). Of these, 133 patients (18%) had grade I, 431 patients (58%) had grade II, and 179 patients (24%) had grade III tumours. Fourteen percent of the 863 patients with clinical recurrence could not be graded, because their tumours were not ductal.

*Survival* Patients with low differentiated tumours had a shorter OS than patients with higher differentiated tumours. The reduction in OS comprised both a reduction of RFI ($P = 0.0001$) and SAR ($P = 0.0001$) of stage II patients, whereas only RFI was effected in stage I patients ($P = 0.02$). The actuarial three year survival rates according to DA I, II, and III were 93%, 89%, and 89% in patients with stage I ($P = 0.06$), and 91%, 76%, and 63% in stage II patients ($P = 0.0001$). Grade III tumours occurred more often in patients with stage II disease than they did in stage I patients ($P < 0.0001$; rank $t$-test).

*Metastatic pattern* Most patients recurred initially in a single site. There were no differences in the number of metastatic sites between groups of patients with tumours of different DA's (Table II). The most common site of first metastasis was bone (36% of all patients with recurrence), followed by recurrence in lung (24%) and local skin (22%). The incidence of visceral metastases was increasing with increasing DA, whereas the distribution of soft tissue and bone metastases was unassociated with DA, both at the time of first recurrence (Figure 1) and at the time of evaluation

**Table I** Distribution of patients with recurrence according to stage, menopausal status, and type of systemic adjuvant therapy

| | $N$ | (%) |
|---|---|---|
| Total number of patients | 863 | (100) |
| Stage I | 228 | (26) |
| Stage II | 635 | (74) |
| Premenopausal | 319 | (37) |
| Postmenopausal | 544 | (63) |
| Systemic adjuvant therapy: | | |
| – none | 479 | (55) |
| – Levamisole | 96 | (11) |
| – Cytotoxic agents | 134 | (16) |
| – Tamoxifen | 154 | (18) |

**Table II** Distribution of patients according to number of metastatic sites at the time of first recurrence and degree of anaplasia of the primary tumour. N (%) indicates the number of patients with recurrence in each group

| | *Degree of anaplasia* | | | | | |
|---|---|---|---|---|---|---|
| | *I* | | *II* | | *III* | |
| *Number of sites* | *N* | (%) | *N* | (%) | *N* | (%) |
| 1 | 100 | (75) | 306 | (71) | 123 | (69) |
| 2 | 22 | (17) | 82 | (19) | 32 | (18) |
| ≥3 | 11 | (8) | 43 | (10) | 24 | (13) |
| TOTAL | 133 | (100) | 431 | (100) | 179 | (100) |

$P=0.38$ (Kruskal–Wallis test).

**Figure 1** Anatomical distribution of metastases at the time of first recurrence according to degree of anaplasia (DA) of the primary tumour. Heights of the columns represent occurrence (%) of metastases in relation to the total number of patients (N) in each stratum.

(Figure 2). In patients with a single site of *first recurrence*, both recurrences of OLN and liver were more common in patients with DA = III tumours than in patients with tumours of lower DA's ($P<0.05$; data not shown). Moreover, in patients with a single site of metastases *at the time of evaluation*, only liver metastases occurred more often in patients with tumours of higher DA ($P<0.05$; data not shown).

*Temporal relations* Two hundred and forty-eight (33%) of the 743 patients with clinical recurrence had their first recurrence within the first year after mastectomy. As expected, there was a significant trend for patients with tumours in higher DA to have recurrence earlier than patients with tumours of lower DA ($P=0.00002$). The anatomical distribution of metastases among patients recurring within the first year after mastectomy is presented in Table III: OLN recurrences and liver metastases occurred more often among patients with DA = III tumours compared to patients with primary tumours of lower grades ($P<0.05$).

**Figure 2** Anatomical distribution of metastases at the time of follow up according to degree of anaplasia (DA) of the primary tumour. For interpretation, see legend to **Figure 1**.

**Table III** Distribution of patients with recurrence within the 1st year after mastectomy according to degree of anaplasia and anatomical site of recurrence. N indicates the total number of patients in each group

| | *Degree of anaplasia* | | |
|---|---|---|---|
| | *I* *N=32* | *II* *N=143* | *III* *N=73* |
| Local skin | 6 | 33 | 19 |
| Other skin | 2 | 6 | 7 |
| Regional lymph nodes | 4 | 25 | 13 |
| Other lymph nodes | 0 | 8 | 13* |
| Contralateral breast | 2 | 9 | 3 |
| Bone | 10 | 58 | 13 |
| Lung | 6 | 27 | 19 |
| Pleura | 2 | 17 | 9 |
| Liver | 1 | 21 | 14* |
| Brain | 1 | 3 | 2 |

*$P<0.05$ (rank *t* test).

*The progression time* (I.e., interval between first and subsequent distant metastases) was shorter for patients with grade II and III tumours than for patients with grade I tumours. Thus, although the differences are not statistically significant, more than 40% of the grade II and III patients developed multiple distant metastases within 3 years compared to 21% of the grade I patients. There were no differences in type of adjuvant treatment and treatment of advanced disease among patients with different DA ($P=0.25$) (Table IV).

*Steroid receptor data*

The ER content of the primary tumour was determined in 110 of the 863 patients; 35 patients (32%) had $<10\,\text{fmol mg}^{-1}$, 44 patients (40%) had $10–99\,\text{fmol mg}^{-1}$, and 31 patients (28%) had $\geq 100\,\text{fmol ER mg}^{-1}$ cytosol protein. The PgR content was measured in 67 of the patients with recurrence; 29 patients (43%) had $<10\,\text{fmol mg}^{-1}$, 21 patients (31%) had $10–99\,\text{fmol mg}^{-1}$, and 17 patients (25%) had $\geq 100\,\text{fmol PgR mg}^{-1}$ cytosol protein.

*Survival* Prolonged RFI ($P=0.001$), SAR ($P=0.0050$), and OS ($P=0.0004$) were associated with increasing ER concentrations. The same pattern applied to PgR determined patients with respect to SAR ($P=0.0062$) and OS ($P=0.0554$), but not to RFI ($P=0.2155$). (All *P* values derive from log rank tests: low *vs.* intermediate *vs.* high SR content, degree of freedom: 2). The SR content was comparable in patients with stage I and II tumours (ER: $P=0.82$; PgR: $P=0.42$; rank *t*-test).

*Metastatic pattern* The ER and PgR content of the primary tumour did not predict the number of metastatic sites either at the time of first recurrence ($P=0.47$ and $P=0.35$, respectively) or at the time of follow up ($P=0.44$ and $P=0.10$, respectively). (*P* values derive from rank *t*-tests: low SR content *vs.* intermediate or high SR content).

The anatomical distribution of metastases at the time of first recurrence is presented according to SR status in Figures 3 and 4. There was a propensity for receptor positive tumours to recur in bone ($P<0.05$), while ER negative tumours more often recurred in lung, liver, and brain. When the ER data were analyzed semiquantitatively, it was found that while the incidence of contralateral breast tumours increased with increasing ER concentrations, visceral metastases occurred more often among patients with low ER content (Table V). These differences were also found at the time of evaluation. The anatomical distribution of metastases according to PgR content was in agreement with the ER data except for contralateral breast and lung (small numbers). Thus, although the numbers are small, the data show a tendency for tumours with high PgR content to recur in bone, and with low PgR content to recur in the liver. The

**Table IV** Distribution of patients with a single distant site of recurrence according to the degree of anaplasia, type of therapy, and proportions of patients with progression within 3 years after recurrence

| *Degree of anaplasia* | *Total no. of patients* | *Type of therapy* | | | | *Three-year actuarial cumulated proportion of patients with progression (%)* |
|---|---|---|---|---|---|---|
| | | *ET*[a] | *CT*[b] | *ET+CT* | *Other* | |
| I | 74 | 29 | 11 | 25 | 9 | (21) |
| II | 230 | 85 | 18 | 91 | 36 | (42) |
| III | 89 | 37 | 7 | 29 | 16 | (46) |

[a]ET = endocrine therapy; [b]CT = chemotherapy.

**Figure 3** Anatomical distribution of metastases at the time of first recurrence according to oestrogen receptor (ER) status of the primary tumour. For interpretation, see legend to **Figure 1**.

**Figure 4** Anatomical distribution of metastases at the time of first recurrence according to progesterone receptor (PgR) status of the primary tumour. For interpretation, see legend to **Figure 1**.

anatomical distribution of metastases was not associated with SR status in the group of patients recurring within the first year after mastectomy, and among patients with only one site of recurrence.

Thirty-seven (34%) of the ER and 23 (34%) of the PgR determined patients had bone metastases. More than 75% of these were located in the spine, and metastases were confined to a single bone region in more than half of the patients. The extent and location of bone metastases was not associated with the SR content (Table VI). Osteolysis, which was the most dominating radiographic morphology, occurred in 76% of the patients with bone metastases; osteosclerosis in 24%, and mixed bone metastases in 14% of the patients. Osteolytic metastases were found more often among patients with low or intermediate ER content than among patients with high ER content ($P<0.05$).

*Progression time* The proportion of patients with a single distant recurrence who subsequently developed other distant metastases is presented according to SR content in Table VII. The proportions are actuarial percentages of patients progressing within 2 years. The proportion increased with both decreasing ER ($P=0.012$) and PgR content ($P=0.003$). The treatment of these patients was not dependent on either the ER content (+/− chemotherapy: $P=0.31$; +/− endocrine therapy: $P=0.24$) or the PgR content ($P=0.25$ and $P=0.58$, respectively).

### Discussion

We have investigated the clinical course of primary and recurrent breast cancer in relation to two established prognostic factors. The aim was first to confirm the impact of the DA and the SR content on prognosis. Secondly, it was the

**Table V** Distribution of patients according to semiquantitative oestrogen receptor receptor (ER) content of the primary tumour and the anatomical sites of metastases at the time of first recurrence and at the time of evaluation. Figures are total number of patients in each group, N, with percentages in parentheses

| | *1st recurrence ER content, fmol mg⁻¹* | | | | | | *Follow up ER content, fmol mg⁻¹* | | | | | |
|---|---|---|---|---|---|---|---|---|---|---|---|---|
| | *<10* | | *10–99* | | *≥100* | | *<10* | | *10–99* | | *≥100* | |
| | *N* | *(%)* | *N* | *(%)* | *N* | *(%)* | *N* | *(%)* | *N* | *(%)* | *N* | *(%)* |
| Number of patients with recurrence | 35 | (100) | 44 | (100) | 31 | (100) | 35 | (100) | 44 | (100) | 31 | (100) |
| *Sites of recurrences:* | | | | | | | | | | | | |
| Skin, local | 9 | (26) | 8 | (18) | 13 | (42) | 10 | (29) | 11 | (25) | 13 | (42) |
| Skin, other | 4 | (11) | 1 | (2) | 3 | (10) | 6 | (17) | 5 | (11) | 4 | (13) |
| Regional lymph nodes | 5 | (14) | 10 | (23) | 1 | (3) | 8 | (23) | 11 | (25) | 4 | (13) |
| Other lymph nodes | 2 | (6) | 5 | (11) | 0 | (0) | 4 | (11) | 6 | (14) | 0 | (0) |
| Contralateral breast | 0 | (0) | 3 | (7) | 3 | (10) | 1 | (3) | 4 | (9) | 5 | (16) |
| Bone | 6 | (17) | 20 | (45) | 11 | (35) | 10 | (29) | 26 | (59) | 14 | (45) |
| Lung | 10 | (29) | 9 | (20) | 6 | (19) | 12 | (34) | 10 | (23) | 6 | (19) |
| Pleura | 2 | (6) | 3 | (7) | 3 | (10) | 4 | (11) | 7 | (16) | 3 | (10) |
| Liver | 7 | (20) | 5 | (11) | 1 | (3) | 10 | (29) | 7 | (16) | 1 | (3) |
| Brain | 2 | (6) | 1 | (2) | 0 | (0) | 6 | (17) | 1 | (2) | 0 | (0) |

**Table VI** Radiographic pattern of bone metastases according to the steroid receptor content of the primary tumour

| | *ER*[a] *content, fmol mg*$^{-1}$ | | | *PgR*[b] *content, fmol mg*$^{-1}$ | | |
|---|---|---|---|---|---|---|
| | *<10* | *10–99* | *>100* | *<10* | *10–99* | *>100* |
| Total no. of patients | 6 | 20 | 11 | 8 | 7 | 8 |
| No. of bone regions with metastases | | | | | | |
| – single | 4 | 11 | 7 | 4 | 3 | 5 |
| – multiple | 2 | 9 | 4 | 4 | 4 | 3 |
| Localization within the skeleton | | | | | | |
| – cranium | 0 | 2 | 3 | 0 | 0 | 0 |
| – columna | 5 | 17 | 8 | 7 | 6 | 5 |
| – pelvis | 2 | 11 | 5 | 4 | 5 | 3 |
| – thorax | 0 | 8 | 4 | 1 | 3 | 2 |
| – extremities | 1 | 8 | 5 | 1 | 4 | 2 |
| Radiographic morphology | | | | | | |
| – osteolysis | 4 | 20 | 4 | 6 | 7 | 6 |
| – osteosclerosis | 1 | 4 | 4 | 2 | 3 | 0 |
| – mixed | 1 | 1 | 3 | 0 | 1 | 3 |

[a]ER = oestrogen receptor; [b]PgR = progesterone receptor.

**Table VII** Steroid receptor content of the primary tumour according to the two-year actuarial cumulated proportion (ACP) of patients, who after initial recurrence in a single distant site developed additional metastatic sites

| *Steroid receptor content, fmol mg*$^{-1}$ | *ER determined patients* | | *PgR determined patients* | |
|---|---|---|---|---|
| | *N*[a] | *ACP %* | *N*[a] | *ACP %* |
| <10 | 21 | 44 | 15 | 50 |
| 10–100 | 28 | 15 | 13 | 14 |
| >100 | 17 | 0 | 13 | 8 |
| *P* (log rank) | 0.012 | | 0.029 | |

[a]N = number of patients.

**Figure 5** Line diagram showing that the mechanisms of action of prognostic factors can be ascribed to chronological and biological circumstances: Biological differences may manifest themselves by variations in pattern of metastases or in growth rate. The parameters used in this study to evaluate biological differences are shown below the dotted line.

purpose to elucidate whether the prognostic effect of these features also reflects different clinical manifestations of the disease, since differences such as metastatic extent and progression rate may reflect basic biological tumour characteristics. Moreover, a connection between a prognostic variable and a certain metastatic pattern and/or growth rate could suggest a specific mechanism of action of the prognostic factor. According to this working hypothesis, which is outlined in Figure 5, the finding of a uniform metastatic pattern and growth rate between prognostic strata indicates that differences in tumour age may play a major role (i.e., chronological prognostic factors).

Histological grading techniques involve subjectivity, and only between 60% and 77% of the results can be reproduced (Stenkvist *et al.*, 1979). Data concerning DA were extracted from the DBCG data base. Tumour grading was not reviewed for the present study, and this may introduce statistical 'noise'. However, from a statistical point of view, random inaccuracies in the measurement of a variable can mask an existing correlation but cannot create an artefactual relationship. Moreover, the result of tumour grading from the DBCG-77 programme has prognostic significance (Andersen *et al.*, 1981; Rank *et al.*, 1987). Thus, despite the multi-institutional basis of the material, the classification according to DA may reflect division of tumours in biological entities. While data on tumour grade are available for 84% of the patients with recurrence, receptor data are available for only 15% of the patients. Although patients with and without receptor measurements were without differences with regard to the occurrence of important prognostic factors, the results of the analysis of grade would appear to be more reliable than those for receptor status.

The study confirms that survival from initial diagnosis is related to both the DA and the SR content of the primary tumour (Schnuerch *et al.*, 1985; Heuson *et al.*, 1977). The reduced survival of patients with either low differentiated or receptor poor tumours applied to a shortening of both RFI

and SAR compared to patients with either high differentiated or receptor rich tumours. This indicates that differences in the status of either DA or SR reflect tumours with different growth rates, and it is further supported by the findings of progression times, which vary with both DA and SR content. The reduced growth rate with increasing SR content may partly be explained by increasing response rates and response durations for patients who received endocrine therapy. However, it seems likely that the SR content also reflects basic growth rate, since the proliferative rate, estimated by the thymidine labelling index, TLI, increases with decreasing SR and increasing DA (Meyer *et al.*, 1986; Adami *et al.*, 1985) and since the TLI is related to prognosis (Strauss *et al.*, 1982).

Patients with different stages of primary breast cancer are supposed to have tumours of different ages (Kamby *et al.*, 1987). Thus, stage of disease may be regarded as a chronological prognostic factor. Higher grades of anaplasia were more common in patients with stage II than stage I tumours. The influence of DA on prognosis may, therefore, also contain an element of tumour chronology. In contrast, since the SR content of the primary tumours was not associated with stage, the impact on survival of SR content does not seem to include differences in tumour age.

In recurrent breast cancer, the prognosis (i.e., the SAR) is among other factors influenced by the anatomical location and the number of recurrences (Vincent *et al.*, 1986; Hietanen *et al.*, 1986). Since SAR in the present study was related to both DA and SR content of the primary tumour, it seems plausible that the influence on prognosis of these factors may be mediated through variations in the pattern of dissemination. However, since the number of anatomical regions with metastases was unassociated with both DA and SR content, it is unlikely that the effect on SAR is mediated through differences in the *extent* of dissemination. When analyzing the *distribution* of metastases, however, it was found that initial signs of poor prognosis also reflect the appearance of metastases in organs where lethality would be greater (i.e., brain, liver, and lung), and that receptor positive patients had a propensity for developing bone metastases compared to receptor negative patients. The increased incidence of liver metastases among patients with low differentiated tumours confirms the findings of Bunting *et al.* (1976) and Coleman & Rubens (1987). Moreover, the literature shows that ER negative tumours preferentially metastasize to viscera (Campbell *et al.*, 1981; Qazi *et al.*, 1984; Singhakowinta *et al.*, 1980; Samaan *et al.*, 1981), while ER positive tumours metastasize to bone (Campbell *et al.*, 1981; Qazi *et al.*, 1984; Singhakowinta *et al.*, 1980; Walt *et al.*, 1976, Stewart *et al.*, 1981; Clark *et al.*, 1987; Williams *et al.*, 1987).

The different metastatic patterns of tumours with varying degrees of tumour differentiation and SR content support experimental data concerning clonal evolution and metastasis. According to these, the appearance of clinical metastases is the end result of a process, where selection, adaptation, and growth of tumour cells in various organs lead to progressive heterogeneity between both the primary tumour and the metastases, and between metastases in various organs (Nowell, 1976; Poste & Fidler, 1980). Thus, the propensity of ER positive tumours to recur in the contralateral breast may suggest a growth lead of these tumours compared to ER negative tumours. Moreover, as the hormonal microenvironment of the contralateral breast is expected to facilitate growth of ER rich tumour cells, tamoxifen treatment may reverse this. In accordance with this view, a reduced incidence of contralateral breast tumours was found in tamoxifen treated patients from the NATO trial (Cuzick & Baum, 1985).

Why SR rich tumours tend to recur more often in bone is not known. Since ER positive tumour cells are capable of inducing osteolysis *in vitro* (Valentin-Opran *et al.*, 1985), these cells may have a survival advantage in the bone system when compared to ER negative cells. In the present study, radiographic osteolysis was more often found in ER negative patients. Since endocrine therapy inhibits the growth of SR rich tumours, and since osteosclerosis may be an indicator of tumour cell response to endocrine therapy (Coombes *et al.*, 1983), the radiographic appearance of osteosclerotic bone metastases in SR positive patients and the appearance of osteolysis in SR negative patients are compatible with the present findings.

The inclusion of tamoxifen treated patients in the current study probably does not introduce bias, because randomization resulted in equal SR contents in tumours of treated and untreated patients, Because of low number of receptor determined patients it was not possible to analyze the metastatic pattern in relation to both the type of adjuvant systemic treatment and SR status at the same time. We have, however, previously reported (Kamby *et al.*, 1988*b*) that failures of adjuvant tamoxifen treatment often involve appearance of lung metastases. This is in agreement with the present results, since ER negative tumours disseminate to lung more often than do ER positive tumours.

In conclusion, it is most likely that the influence on prognosis of tumour grade and SR content works through multiple 'mechanisms'. Of these, biological growth properties such as growth rate and metastatic pattern are probably of greatest importance. The acknowledgement of site specific differences in SR content has implications for rational application of endocrine therapy. Thus, one should not regard patients as either receptor positive or negative, based on primary tumour determinations only. Instead, SR measurements should be performed on metastases in various locations before deciding on treatment.

Supported by a grant from the Danish Medical Research Council no. 12-6006.

### References

ADAMI, H.-O., GRAFFMAN, S., LINDGREN, A. & SALLSTROEM, J. (1985). Prognostic implication of estrogen receptor content in breast cancer. *Breast Cancer Res. Treat.*, **5**, 293.

ANDERSEN, J.A., FISCHERMANN, K., HOU-JENSEN, K. & 8 others (1981). Selection of high risk groups among prognostically favorable patients with breast cancer. *Ann. Surg.*, **194**, 1.

ANDERSEN, K.W., MOURIDSEN, H.T., CASTBERG, Th. & 8 others (1981). Organisation of the Danish adjuvant trials in breast cancer. *Dan. Med. Bull.*, **28**, 102.

BARTOLUCCI, A.A. (1984). Estimations and comparisons of proportions. In *Cancer Clinical Trials – Methods and Practice*, Buyse *et al.* (eds) p. 337. Oxford University Press: Oxford.

BLOOM, H.J.G. & RICHARDSON, W.W. (1957). Histological grading and prognosis in breast cancer (a study of 1409 cases of which 359 have been followed for 15 years). *Br. J. Cancer*, **11**, 359.

BRINKLEY, D. & HAYBITTLE, J.L. (1975). The curability of breast cancer. *Lancet*, **ii**, 95.

BROSS, I.D.J. (1954). Is there an increased risk? *Fed. Proc.*, **ii**, 815.

BUNTING, J.S., HEMSTED, E.H. & KREMER, J.K. (1976). The pattern of spread and survival in 596 cases of breast cancer related to clinical staging and histological grade. *Clin. Radiol.*, **27**, 9.

CAMPBELL, F.C., BLAMEY, R.W., ELSTON, C.W. & 4 others (1981). Quantitative oestradiol receptor values in primary breast cancer and response of metastases to endocrine therapy. *Lancet*, **ii**, 1317.

CLARK, G.M., SLEDGE, G.W. JR., OSBORNE, C.K. & McGUIRE, W.L. (1987). Survival from first recurrence: Relative importance of prognostic factors in 1,015 breast cancer patients. *J. Clin. Oncol.*, **5**, 55.

COLEMAN, R.E. & RUBENS, R.D. (1987). The clinical course of bone metastases from breast cancer. *Br. J. Cancer*, **55**, 61.

COOMBES, R.C., DADY, P., PARSONS, C. & 4 others (1983). Assessment of response of bone metastases to systemic treatment in patients with breast cancer. *Cancer*, **52**, 610.

CUZICK, J. & BAUM, M. (1985). Tamoxifen and contralateral breast cancer. *Lancet*, **i**, 282.

EORTC BREAST CANCER COOPERATIVE GROUP (1980). Revision of the standards for the assessments of hormone receptors in human breast cancer. *Eur. J. Cancer*, **16**, 1513.

HEUSON, J.C., LONGEVAL, E., MATTHEIEM, W.H., DEBOEL, M.C., SYLVESTER, R.J. & LECLERCQ, G. (1977). Significance of quantitative assessment of estrogen receptors for endocrine therapy in advanced breast cancer. *Cancer*, **39**, 1971.

HIETANEN, P., MIETTINEN, M. & MAEKINEN, J. (1986). Survival after first recurrence in breast cancer. *Eur. J. Cancer Clin. Oncol.*, **22**, 913.

KAMBY, C., ROSE, C., EJLERTSEN, B. & 5 others (1987). Stage and pattern of metastases in patients with breast cancer. *Eur. J. Cancer Clin. Oncol.*, **23**, 1925.

KAMBY, C., ROSE, C., EJLERTSEN, B. & 5 others (1988). Adjuvant systemic treatment and the pattern of recurrences in patients with breast cancer. *Eur. J. Cancer Clin. Oncol.*, **24**, 439.

KLEINBAUM, D.G., KUPPER, L.L. & MORGENSTERN, H. (1982). Epidemiologic research – Principles and quantitative methods. Ch. 17, p. 320. Van Nostrand Reinhold Company: New York.

MEYER, J.S., PREY, M.U., BABCOCK, D.S. & McDIVITT, R. (1986). Breast carcinoma cell kinetics, morphology, stage, and host characteristics. *Lab. Invest.*, **54**, 41.

NOWELL, P.C. (1976). The clonal evolution of tumour cell populations. *Nature*, **194**, 23.

PETO, R., PIKE, M.C., ARMITAGE, P. & 7 others (1977). Design and analysis of randomized clinical trials requiring prolonged observation of each patient. II. Analysis and examples. *Br. J. Cancer*, **35**, 1.

POSTE, G. & FIDLER, I.J. (1980). The pathogenesis of cancer metastasis. *Nature*, **283**, 139.

QAZI, R., CHUANG, J.-L.C. & DROBYSKI, W. (1984). Estrogen receptors and the pattern of relapse in breast cancer. *Arch. Intern. Med.*, **144**, 2365.

RANK, F., DOMBERNOWSKY, P., BANG JESPERSEN, N.C., VESTERGAARD PEDERSEN, B. & KEIDING, N. (1987). Histological malignancy grading of invasive ductal breast carcinoma. *Cancer*, **60**, 1299.

SAMAAN, N.A., BUZDAR, A.U., ALDINGER, K.A. & 4 others (1981). Estrogen receptor: A prognostic factor in breast cancer. *Cancer*, **47**, 554.

SCARFF, R.W. & TORLONI, H. (1968). Histological typing of breast tumours. International histological classification of tumours. WHO Geneva, p. 2.

SINGHAKOWINTA, A., SAUNDERS, D.E., BROOKS, S.C., SAMAL, B. & VAITKEVICIUS, V.K. (1980). Clinical application of estrogen receptor in breast cancer. *Cancer*, **46**, 2932.

STENKVIST, B., WESTMAN-NAESER, S., VEGELIUS, J. & 4 others (1979). Analysis of reproducibility of subjective grading systems for breast carcinoma. *J. Clin. Pathol.*, **32**, 979.

STEWART, J.F., KING, R.J.B., SEXTON, S.A., MILLIS, R.R., RUBENS, R.D. & HAYWARD, J.L. (1981). Oestrogen receptors, sites of metastatic disease and survival in recurrent breast cancer. *Eur. J. Cancer*, **17**, 449.

STRAUSS, M.J., MORAN, R., MULLER, E. & WOTIZ, H.H. (1982). Estrogen receptor heterogeneity and the relationship between estrogen receptor and the tritiated thymidine labelling index in human breast cancer. *Oncology*, **39**, 197.

VALENTIN-OPRAN, A., EILON, G., SAEZ, S. & MUNDY, G.R. (1985). Estrogens and antiestrogens stimulate release of bone resorbing activity by cultured human breast cancer cells. *J. Clin. Invest.*, **75**, 726.

VINCENT, M.D., POWLES, T.J., SKEET, R. & 6 others (1986). An analysis of possible prognostic features of long term and short term survivors of metastatic breast cancer. *Eur. J. Cancer Clin. Oncol.*, **22**, 1059.

WALT, A.J., SINGHAKOWINTA, A., BROOKS, S.C. & CORTEZ, A. (1976). The surgical implications of estrophile protein estimations in carcinoma of the breast. *Surgery*, **80**, 506.

WILLIAMS, M.R., TODD, J.H., ELLIS, C.S. & 6 others (1987). Oestrogen receptors in primary and advanced breast cancer: An eight year review of 704 cases. *Br. J. Cancer*, **55**, 67.